# The impact of health in all policies on urban innovation: a mediation analysis of human capital accumulation using panel data from 271 cities in China

**DOI:** 10.3389/fpubh.2025.1663598

**Published:** 2025-09-24

**Authors:** Jun Wang, Shuangyu Zhao, Yijun Liu

**Affiliations:** ^1^China Institute of Health, Renmin University of China, Beijing, China; ^2^Center for Health Policy Research and Evaluation, Renmin University of China, Beijing, China; ^3^School of Public Administration and Policy, Renmin University of China, Beijing, China

**Keywords:** health in all policy, HiAP, urban innovation, human capital accumulation, China

## Abstract

**Introduction:**

This study investigates the impact of Health in All Policies (HiAP) on urban innovation within the context of China. Building on migration theory, innovation ecosystem theory, and the country's institutional context, we identify human capital accumulation as a key mediating factor.

**Methods:**

Using a panel dataset of 271 cities from 2013 to 2021, we use the two-way fixed effects regressions, the Difference-in-Differences (DID) model, and the stepwise regression analysis method.

**Results:**

We find that HiAP significantly enhances urban innovation with the magnitude of its effect varying across city size, administrative level and geographic region. Furthermore, human capital accumulation mediates the relationship between HiAP and innovation outcomes.

**Discussion:**

These results underscore the importance of HiAP in promoting innovation through human capital development and offer practical policy implications for advancing sustainable urban development.

## Highlights

- HiAP boosts urban innovation in 271 Chinese cities (2013–2021).
- Human capital mediates HiAP's effect on urban innovation.
- Effects vary by city size, administrative level, and region.
- Combines flow-stock views to assess reserves and renewal of human capital accumulation.
- Builds a tripartite health-human capital-innovation framework.

## 1 Introduction

In the era of the knowledge economy, urban innovation plays a pivotal role in globalization by driving economic growth, improving social wellbeing, and advancing sustainable development. Traditional studies on innovation have largely emphasized explicit factors such as technology and capital (72), while often neglecting the broader systemic influence of public policy. Recent scholarship increasingly recognizes urban innovation as a complex, multidimensional process shaped not only by technological progress and financial investment but also by institutional frameworks, social structures, and human capital accumulation (1). This highlights the need to examine how health policies can indirectly enhance innovation capacity by shaping pathways for human capital development. Against this backdrop, Health in All Policies (HiAP) has emerged as a key paradigm in global health governance, characterized by its emphasis on collaborative, cross-sectoral policy integration. The central premise of HiAP is the incorporation of health objectives into diverse domains such as economic development, environmental sustainability, and social governance. By systematically accounting for health considerations in policymaking and implementation, HiAP facilitates the efficient allocation of social resources, improves population health, strengthens human capital for innovation, and ultimately fosters health equity and sustainable urban development.

The relationship between health and innovation is not coincidental but reflects deep institutional linkages between health policy frameworks and urban development trajectories. Health is not only a fundamental aspect of individual wellbeing but also a crucial element of human capital, directly influencing workforce productivity, creativity, and overall socioeconomic contributions (74). Insights from health economics and growth theory suggest that population health, at both individual and societal levels, plays a vital role in national economic advancement, with health-related human capital significantly contributing to income generation, personal welfare, and economic progress (2). As a result, health capital is now widely recognized as a core element of human capital in modern socioeconomic contexts. For instance, deficiencies in childhood health can permanently hinder human capital formation by impeding cognitive development and educational achievement, while the health status of adults directly affects labor productivity through illness-related absenteeism and reduced on-the-job performance (3). Furthermore, adopting a HiAP approach can enhance the social determinants of health, fostering a supportive environment for urban innovation. Studies suggest that integrating health and development policies can drive urban revitalization and cultivate a more conducive atmosphere for innovation (75). Recent research by Olson et al. (4) has demonstrated that health policies yield positive spillover effects, as public health measures stimulate corporate innovation by enhancing health outcomes, boosting productivity, reducing absenteeism, and expanding the workforce (4). Empirical evidence from various countries, such as the United Kingdom, Finland, and Australia, highlights the structural enhancement of human capital quality resulting from health policies, ultimately fueling urban innovation (5).

However, existing scholarship on the influence of health policies on urban innovation has several key limitations that need to be addressed. Firstly, current urban innovation literature often overlooks the role of health determinants as endogenous drivers, instead treating them as exogenous variables. This approach creates a theoretical gap that hinders policymakers from recognizing the strategic value of investments in health, contrary to the health-in-all-policies approach advocated by organizations like the WHO. Moreover, there is a lack of quantitative analysis on the long-term effects of HiAP, with most studies focusing on immediate economic outcomes such as healthcare cost containment and pandemic response efficacy, while neglecting the long-term innovation benefits from human capital accumulation. Secondly, incomplete mediation frameworks in existing research limit causal inference. Current studies tend to emphasize direct correlations between human capital and innovation, without fully developing theoretical models that position human capital as a mechanism for policy transmission. Thirdly, the regional heterogeneity of health policies' impact on urban innovation is often overlooked. Many analyses rely on national or provincial data, masking variations in resource endowments, policy implementation, and economic structures among cities. Lastly, empirical research lacks support for policy designs that balance multiple value orientations. There is a scarcity of robust examinations on whether municipal health governance, human capital resources, and technological innovation capacities can be effectively coordinated to achieve policy integration that addresses equity, efficiency, and sustainability imperatives. Addressing these limitations is crucial for developing evidence-based policies that promote urban innovation while prioritizing health outcomes.

This study systematically examines the influence of HiAP on urban innovation and its underlying mechanisms through a comprehensive blend of theoretical frameworks and empirical analyses. The research offers fresh theoretical and empirical perspectives on the interconnected relationship involving health governance, human capital accumulation, and innovation ecosystems. In practical terms, the study translates the national strategic priority of “health-driven innovation” into actionable insights, endorsing policy coherence between China's Healthy City initiatives and innovation-centered urban development strategies. Moreover, the study puts forth practical HiAP optimization strategies to propel sustainable urban progress. The inquiry focuses on four key research inquiries: (1) Does the implementation of HiAP have a statistically significant positive effect on patent-based urban innovation? (2) Does the human capital accumulation act as a mediator between HiAP and urban innovation? (3) Do the effects of policies exhibit distinct urban heterogeneity across regions? To explore the impact of HiAP on urban innovation, this research constructs a panel dataset encompassing 271 Chinese cities over the period from 2013 to 2021.

The paper is structured as follows. First, we review the existing literature on the impact of HiAP on urban innovation and its underlying mechanisms, and propose hypotheses. Second, we explain the measurement of variables such as HiAP, human capital accumulation, and urban innovation, as well as the data sources, and propose empirical models. Third, we present the empirical results and robustness tests, additionally focusing on the impact of the five dimensions of HiAP on the dependent variable. Fourth, we analyze the mediating effects of human capital accumulation and urban heterogeneity. Finally, we summarize the conclusions of the study and put forward policy implications and research limitations.

## 2 Theoretical background and research hypotheses

### 2.1 Health in all policies (HiAP)

#### 2.1.1 Conceptual evolution

The origins of HiAP can be traced back to the nineteenth-century public health movement (6). Early expressions of the concept appeared in the Alma-Ata Declaration (76) and the Ottawa Charter (77), which underscored the significance of social determinants of health, conducive environments for health promotion, and cross-sectoral collaboration. The 1988 Adelaide Statement by the World Health Organization introduced a strategic framework for governments to incorporate health considerations into policy planning. In 2006, HiAP was formally introduced as a distinct policy framework, with the goal of integrating health considerations into all policies through intersectoral collaboration to enhance population health and health equity (7). Another pivotal moment in the advancement of the HiAP concept occurred during the 8th Global Conference on Health Promotion (8GCHP) in Helsinki, Finland, in 2013. At this event, definitions and frameworks pertaining to HiAP were introduced. In 2015, the World Health Assembly endorsed the WHO Framework for Country Action on Intersectoral Action for Health and Health Equity, which articulated the essential components for implementing HiAP. Table 1 summarizes the conceptual evolution of HiAP. Importantly, HiAP extends far beyond healthcare policy, embedding health considerations across the entire economy—a feature that is central to its impact on innovation.

**Table 1:** Conceptual evolution of Health in All Policies (HiAP).

**Period**	**Key document**	**Theoretical contribution**
1972	Report by the economic council of Finland	The launch of the North Karelia project
1974	Lalonde report by the Canadian ministry of health	Five strategies and 74 actions aimed at promoting HiAP
1978	Alma-ata declaration	Early intersectoral health concepts
1986	Ottawa charter	Early manifestations of the HiAP concept
1988	Adelaide statement by the world health organization	A strategic framework for institutionalization of policy integration
2006	“Health in all policies: prospects and potentials publication” of Finland	Formally establishing the concept of HiAP
2011	Rio political declaration on social determinants of health	Underscored HiAP in Article 7
2013	8GCHP in Helsinki, Finland	Formal definition of HiAP as impact-assessment system
2015	WHO framework for country action	Standardized implementation components

#### 2.1.2 Current research status

Current research on HiAP has focused on three main areas. First, studies have underscored the importance of integrating health goals across different policy sectors using cross-sectoral governance mechanisms. Research has shown that effective intersectoral coordination is crucial for addressing complex global health issues and promoting Sustainable Development Goals (SDGs). Successful implementation relies on strong governance structures, accountability measures, and engaging stakeholders (8). Second, advancements in Health Impact Assessment (HIA) have positioned it as a practical tool for implementing the HiAP approach and as a means to assess health equity outcomes resulting from policies. For instance, Green (9) demonstrates the effectiveness of HIA adoption in Wales, though sustaining its impact requires supporting conditions such as institutional capacity building and workforce development (9). Third, comparative analyses of policies have shown that HiAP is predominantly concentrated in advanced economies, with 14 jurisdictions in Europe, North America, and Oceania having established HiAP frameworks (10). For example, South Australia's innovative HiAP model, developed in collaboration with the World Health Organization, exemplifies comprehensive integration through governance reforms, Health Lens Analysis techniques, and accountability measures, ultimately improving the wellbeing of the population.

Despite these advances, current HiAP research faces several limitations. First, it predominantly focuses on health impact assessment, health equity evaluation, and policy process analysis, neglecting crucial connections between HiAP frameworks and urban innovation. This oversight impedes practical responses to the strategic policy objectives of health prioritization, coordinated innovation, and sustainable development. Second, existing evaluations heavily rely on qualitative analyses, lacking standardized quantitative metrics, longitudinal datasets, and robust theoretical frameworks, especially in assessing policy-induced health externalities. Third, HiAP implementation and research disproportionately concentrate on developed economies, neglecting the challenges faced in developing nations due to resource constraints and institutional barriers. This necessitates urgent exploration of context-specific implementation strategies that consider local sociocultural dynamics and governance capacities.

### 2.2 Urban innovation and HiAP

#### 2.2.1 Urban innovation

In the era of the knowledge economy, innovation plays a pivotal role in driving economic growth, enhancing urban competitiveness, and advancing technology (11). Urban innovation is widely acknowledged as a crucial factor for metropolitan competitiveness and resilience, serving as a measurable gauge of a city's ability to engage in creative problem-solving and technological adaptation, which are directly linked to long-term economic viability. Urban innovation measurement methodologies are commonly classified into two main approaches: single-indicator assessments and composite indicator systems. Traditional metrics typically emphasize three key dimensions: innovation output (measured by patent grants), input intensity (quantified through R&D expenditure), and efficiency ratios. While these conventional parameters effectively capture fundamental innovation capacity, they do not adequately address the systemic challenges and hidden costs inherent in urban innovation processes (12). In addition to these single-indicator methods, comprehensive evaluation frameworks have gained methodological prominence. For instance, the Innovative City Index utilizes a hierarchical structure comprising five primary domains supported by thirty sub-indicators, encompassing aspects like innovation governance, technological innovation, and innovation-driven growth (13). This framework not only quantifies multidimensional innovation capacity but also serves as a policy tool for promoting sustainable socio-economic development through science and technology initiatives.

#### 2.2.2 The impact of HiAP on urban innovation

The relationship between HiAP and urban innovation remains debated, with mixed findings in the literature. Some scholars argue for a positive correlation, contending that health plays a pivotal role in human capital accumulation and sustainable development (14). They posit that HiAP enhances human capital by bolstering residents' health, thereby indirectly fostering regional innovation capacities. Conversely, other scholars suggest a negative association between HiAP and urban innovation. They highlight challenges such as resource competition, intricate policy processes, and obstacles in interdepartmental implementation inherent in HiAP, which could impede urban innovation progress (15). Given the diverse contextual factors across countries, it is imperative to investigate the HiAP-urban innovation nexus within the Chinese context, an area that remains underexplored in current scholarship.

This study tends to support the positive relationship between HiAP and urban innovation in China. According to the Innovation Ecosystem Theory, significant innovations typically arise from complex networks rather than isolated factors. The study suggests that breakthrough innovations result from collaborative interactions within diverse innovation ecosystems involving various stakeholders such as private sector companies, government entities, academic institutions, universities, and non-governmental organizations. These interactions facilitate knowledge sharing and resource complementarity, leading to the reconfiguration of innovation pathways through dynamic interorganizational dependencies (16). The HiAP framework facilitates intersectoral synergies by aligning health goals with cross-sector governance, thereby stimulating systemic innovation within urban ecosystems, particularly in knowledge and output innovations. Firstly, the implementation of HiAP requires knowledge exchange among multiple stakeholders and technical collaborations between organizations, fostering technological advancements and creating a robust knowledge base for patent applications. Secondly, policy incentives within the HiAP framework, such as financial backing and tax advantages, can directly boost innovation endeavors, translating into tangible patent outcomes (17). Furthermore, the collaborative efforts across sectors can optimize resource utilization, lower innovation expenses, and ultimately enhance urban innovation capabilities (18). Consequently, this study posits the following initial hypothesis:

H1: the implementation of HiAP will increase urban innovation.
H1a: the implementation of HiAP will increase urban knowledge innovation (the number of application patents, Patap).
H1b: the implementation of HiAP will increase urban product innovation (the number of authorization patents, Patau).

One of the reasons for the inconsistent effects of HiAP on urban innovation is heterogeneity. Specifically, there may be differences in urban innovation capacity among cities with different population sizes, administrative levels, and geographical locations. Firstly, larger cities tend to have greater human capital accumulation, enhancing the potential for HiAP to drive innovation. Additionally, densely populated areas benefit from economies of scale, attracting high-tech talent and resources, which smaller cities struggle to replicate due to resource dispersion (19). Secondly, the influence of HiAP on urban innovation varies across administrative hierarchies. Cities at higher administrative levels leverage their statutory authority and fiscal capabilities to implement the policy effectively (20). Thirdly, geographical location plays a crucial role in shaping policy outcomes. Regional disparities impact the efficacy of HiAP in fostering urban innovation. Coastal eastern regions with advanced economic structures and global integration demonstrate heightened policy effectiveness. Furthermore, the contrasting economic landscapes between southern and northern cities result in divergent impacts of HiAP on urban innovation (21). The influence of HiAP on urban innovation displays variations based on factors such as population size, administrative hierarchy of cities, and geographical positioning.

H2: the impact of HiAP on urban innovation varies, with differences observed in terms of population size, administrative level of cities, and geographical location.
H2a: the impact of HiAP on urban innovation exhibits significant variations in the population size.
H2b: the impact of HiAP on urban innovation exhibits significant variations in the administrative hierarchy.
H2c: the impact of HiAP on urban innovation exhibits significant variations in the geographical location, among east, central, and west city distribution.
H2d: the impact of HiAP on urban innovation exhibits significant variations in the geographical location, between southern and northern cities.

### 2.3 HiAP, urban innovation and human capital accumulation

#### 2.3.1 Human capital accumulation

Schultz (22) first introduced the concept of human capital accumulation, emphasizing the ability to acquire knowledge, skills, and other valuable attributes through education, training, migration, and health investment to enhance productivity and innovation. Various metrics gauge human capital accumulation, such as the Human Development Index (HDI), World Bank Human Capital Index (HCI), PISA, and PIAAC, which assess students' cognitive abilities. Scholars typically evaluate human capital accumulation through health, education, and migration. First, health indicators like life expectancy, healthy life years, public health investment, and the health status of the working-age population are commonly used to measure health capital (23). Second, education level is a key proxy for human capital, with metrics including average years of education per capita, higher education density, and R&D personnel proportion (24). Third, migration indicators like net migration rate, permanent resident to registered population ratio, and proportion of high-skilled immigrants reflect human capital in cities (25). Despite efforts to capture human capital's multifaceted nature, there is a consensus in the academic community on the need for improved indicators to better represent its essence.

#### 2.3.2 The mediating role of human capital accumulation

**Figure 1:**
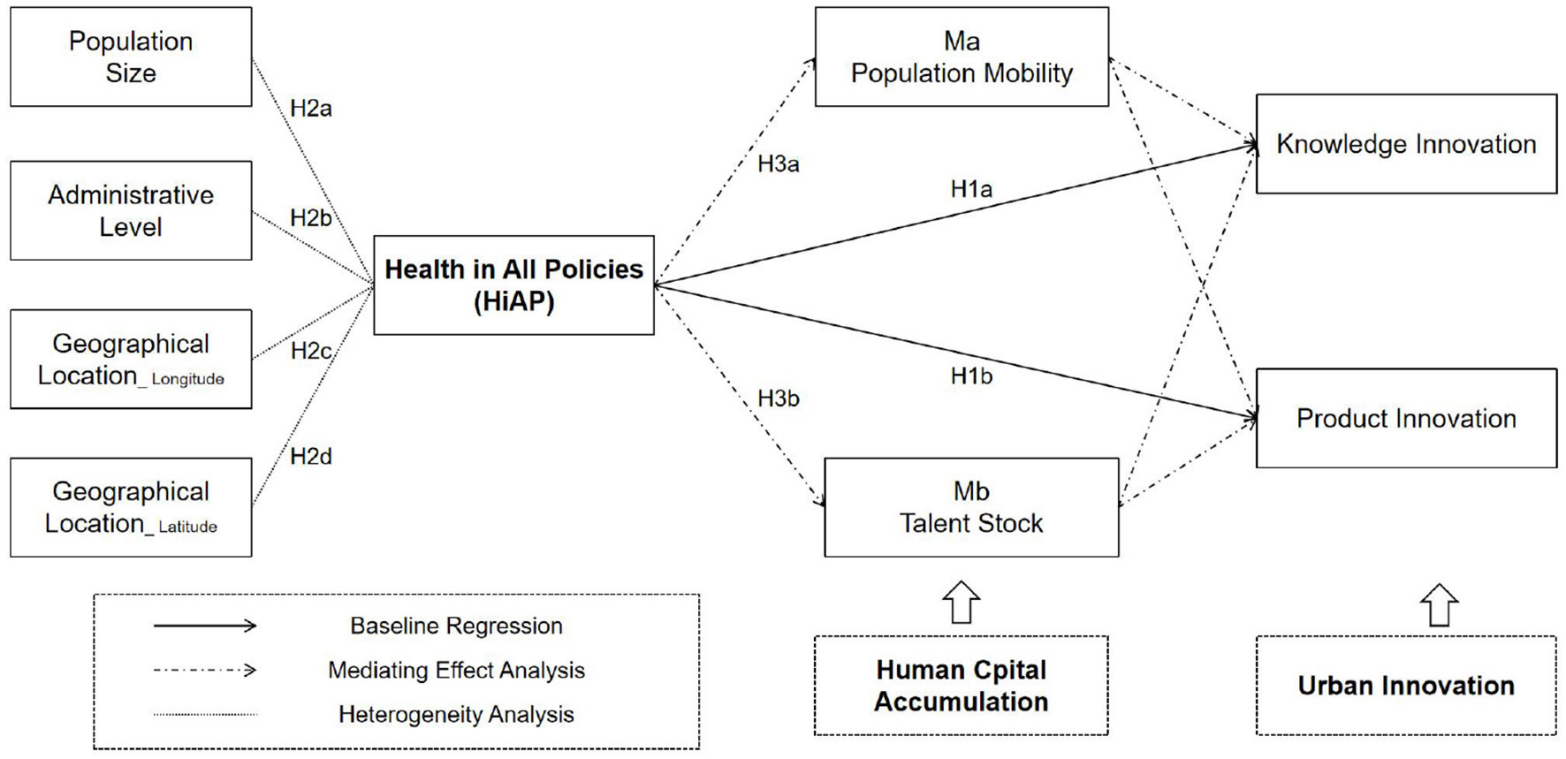
Theoretical model.

As mentioned above, the impact of HiAP on urban innovation is currently ambiguous. The complexity of this relationship stems from the absence of a straightforward direct connection between HiAP and urban innovation. Rather, HiAP often necessitates conversion into specific forms of human capital, which are linked to urban innovation. While various studies have started to investigate mediating factors, the role of human capital as a mediator in translating policy effects into urban innovation has been widely acknowledged. Nonetheless, limited research has approached this issue through the lens of Health in All Policies. For instance, human capital can act as a mediating factor in areas such as public service provision, the promotion of civilized cities, and the implementation of new urbanization initiatives, thereby impacting cities' innovation capabilities (13). Further exploration is warranted to delve into the mediating effect of human capital accumulation on the relationship between HiAP and urban innovation. The theoretical model for this study is illustrated in Figure 1.

This study proposes that the implementation of HiAP can positively impact urban innovation by enhancing human capital accumulation. HiAP is believed to boost urban innovation through two main mechanisms. Firstly, HiAP promotes organized labor mobility and concentration, thereby bolstering urban innovation capacities. According to migration theory, factors such as economic opportunities, cultural amenities, and social services in destination areas play a crucial role in attracting labor at a broader structural level (26). HiAP underscores the integration of public health considerations into various policy domains including the economy, environment, education, and transportation, with evaluation frameworks prioritizing ecological factors, social welfare, and healthcare infrastructure. By addressing health-related social determinants, HiAP contributes to the creation of livable communities that improve quality of life and wellbeing, making them attractive for skilled workforce migration and human capital concentration. Secondly, HiAP enriches talent pools by establishing comprehensive health support systems across the life course to optimize human capital development. For example, investments in early childhood health have been shown to enhance the quality of the future workforce, as research indicates that early childhood interventions (ECIs) positively influence educational achievements in later stages of life (27). Furthermore, education policies focusing on health have significantly reinforced the foundational development of adolescents, with studies illustrating that initiatives such as school nutrition enhancement programs enhance cognitive abilities and physical fitness (28). These efforts systematically contribute to higher rates of students completing advanced education and expanding the pool of high-quality talent.

Based on this, the following hypothesis is made:

H3: human capital accumulation (HCA) plays a mediating role in the relationship between the implementation of HiAP and urban innovation.

H3a: the implementation of HiAP enhances urban innovation by improving population mobility (HCA1).

H3b: the implementation of HiAP enhances urban innovation by improving talent stock (HCA2).

## 3 Variables and model specification

### 3.1 Data sources

To assess the influence of Health in All Policies (HiAP) on urban innovation, this study utilizes a panel dataset comprising 271 prefecture-level and higher cities in China from 2013 to 2021 as the research sample. The specified data sources are as follows:

#### 3.1.1 Dependent variable

Patent data are obtained from the China National Intellectual Property Administration (CNIPA), specifically through the China Innovation Research Database (CIRD) of the Chinese Research Data Services Platform (CNRDS).

#### 3.1.2 Independent variables

HiAP indicators are primarily sourced from the China City Statistical Yearbooks (2013–2022), with missing values supplemented by official municipal statistical bulletins.

#### 3.1.3 Mediating variables

Human capital metrics derive from the China City Statistical Yearbooks and China Labor Statistical Yearbooks.

#### 3.1.4 Control variables

Digital finance data are drawn from the Peking University Digital Financial Inclusion Index (PKU-DFI), jointly developed by the Institute of Digital Finance at Peking University and Ant Group. This index quantifies financial service dimensions (breadth, depth, and regional disparity) across 31 provinces, 337 prefecture-level cities, and approximately 2,800 counties during 2011–2023 (29) Digital infrastructure metrics (optical fiber route length and mobile base station density) are extracted from provincial statistical yearbooks (2013–2022), while other variables are compiled from the China City Statistical Yearbooks (2013–2022), with data gaps filled using prefecture-level statistical reports.

#### 3.1.5 Alternative dependent variable

The China City and Industrial Innovation Index (CIIC) was jointly released by the Fudan-TSE Research Institute of Innovation and Digital Economy (RIDE) and Fudan Institute of Industrial Development (FIND).

#### 3.1.6 Alternative independent variable

Based on the announcement by China's National Health Care Office regarding the pilot project of healthy cities, a total of 38 cities were selected as the treatment group for healthy city construction (30).

The missing data in certain years were filled using the data from Statistical Yearbook of Prefecture level Cities from official websites, and then using the linear interpolation approach, resulting in a total of 2,196 observations.

### 3.2 Variable definition and calculation

#### 3.2.1 Dependent variable

Patents serve as a crucial metric for assessing a region's innovation progress. This study suggests that innovation comprises knowledge innovation and product innovation. Knowledge innovation involves generating and accumulating novel knowledge and technologies, marking the initial phase of technological advancements. In contrast, product innovation concentrates on transforming technological progress into tangible applications and marketable products or services. Both forms are integral elements of a holistic innovation framework. Drawing on prior research (31–33), we selected the number of application patents and authorization patents as indicators to represent knowledge innovation and product innovation, respectively, sourced from the China National Intellectual Property Administration.

#### 3.2.2 Independent variable

The independent variable is Health in All Policies (HiAP). This study defines Health in All Policies (HiAP) as a comprehensive and cross-cutting policy framework aimed at incorporating health considerations throughout various governance sectors. Through emphasizing health impact assessments within cooperative governance frameworks, HiAP aims to enhance public health results, diminish health inequalities, and foster fair socioeconomic progress by systematically addressing health factors in decision-making procedures, thereby fostering enduring societal change. Following a Chinese Blue Book (71), we use five dimensions to establish the evaluation index system of the implementation status of HiAP in 289 prefecture level and above cities in the Chinese Mainland, including Health in Economy, Health in Public Service, Health in Environment, Health in Culture, and Health in Medical Care. Table 2 presents the indicators of HiAP index.

**Table 2:** Indicators of Health in All Policies (HiAP).

**First-level Indicators**	**Weight**	**Second-level Indicators**	**Weight**	**Third-level Indicators**
A. Health in economy	0.220	A1. Economic base	0.543	4 indicators
		A2. Life consumption	0.457	6 indicators
B. Health in public service	0.150	B1. Social security	0.471	3 indicators
		B2. Social stability	0.286	2 indicators
		B3. Infrastructure	0.243	5 indicators
C. Health in environment	0.183	C1. Ecological environment quality	0.427	2 indicators
		C2. Pollution control status	0.324	5 indicators
		C3. Environmental infrastructure	0.249	1 indicator
D. Health in culture	0.100	D1. Cultural investment	0.371	2 indicators
		D2. Educational level	0.350	1 indicator
		D3. Cultural infrastructure	0.279	4 indicators
E. Health in medical care	0.347	E1. Medical resource	0.629	5 indicators
		E2. Medical investment	0.371	1 indicator

Among the 41 indicators, two metrics with negative polarity—the urban registered unemployment rate and sulfur dioxide (SO_2_) emissions—are inversely scored, while all others demonstrate positive directional attributes in the evaluation framework.

The evaluation method for HiAP index adopts linear weighting method, embodying the principle of combining subjectivity and objectivity. The first step is to standardize the evaluation indicators to ensure that the maximum values are either 1 or 100. There are m indicators, namely Z_1_, Z_2_ ... Z_m_, with weights of W_1_, W_2_, ... W_m_.

The positive indicator standardization method can be achieved by dividing the indicator value by the maximum value:


(1) $$\begin{array}{c} X_{ij}=\frac{Z_{ij}}{max\;\left(Z_{ij}\right)} \end{array}$$

The standardization method for reverse indicators is:


(2) $$\begin{array}{c} X_{ij}=1-\frac{Z_{ij}}{max\;\left(Z_{ij}\right)} \end{array}$$

The evaluation indicators are based on objective data. Specifically, as a constant evaluation result with continuous data, it is beneficial for comparison between different cities. Also, under the condition of unchanged weights, this index can be compared vertically. In other words, linear weighting method not only achieves comparability of HiAP index between different cities in the same year, but also achieves comparability of the same city in different years, which is conducive to finding gaps and making improvements.

What's more, the weights of each indicator were determined by expert meeting method. According to the annual report on urban health life in China (71), more than 20 experts from relevant fields were invited. After the first round of scoring, the average weight was fed back for the second round of scoring. After three rounds, the weights tended to stabilize.

#### 3.2.3 Mediating variable

The mediating variable is human capital accumulation, assessed bidirectionally through flow-oriented and stock-oriented perspectives. Following the research of human capital (34, 35), we use population mobility (HCA1) and talent stock (HCA2) to reflect human capital accumulation. On the one hand, the population mobility is calculated as the ratio of the difference between the current period resident population and the previous period resident population to the current period household registered population. On the other hand, based on data availability, the urban-level talent stock is quantified using the ratio of the number of students enrolled in regular undergraduate and junior college programs to the household registered population (%). The calculation formulas are as follows:


(3) $$\begin{array}{c} HCA1=PopulationMobility=\\ \frac{Resident\;Population_{Current\;Period}\;-\;Resident\;Population_{Previous\;Period}}{Household\;Registered\;Population_{Current\;Period}}\\ =\;\frac{P_{resident}\;\left(Current\right)-P_{resident}\;\left(Previous\right)}{P_{hukou}\;\left(Current\right)} \end{array}$$

(4) $$\begin{array}{c} HCA2=Talent\;Stock=\;\\ \frac{Number\;of\;Students\;Enrolled\;in\;Regular\;Undergraduate\;and\;Junior\;College\;Programs}{Household\;Registered\;Population\;\%}\;\\ =\;\frac{P_{student}}{P_{hukou}\%} \end{array}$$

Note: ensure that the units of the variables in the numerator and denominator are consistent.

We take the results as the proxy variables of human capital accumulation. The primary advantage of the former indicator lies in its adaptability to the nuances of urbanization in China. By incorporating data on permanent resident population alongside household registration statistics, potential distortions arising from the registration system are mitigated. Moreover, this indicator captures implicit human capital, particularly non-academic skills prevalent among the floating population, which are challenging to quantify using educational metrics. The latter indicator's key strength stems from the international standardization of higher education qualifications, enabling seamless cross-regional comparisons and ensuring robust data availability. By integrating both flow-oriented and stock-oriented perspectives, the assessment of human capital accumulation offers a comprehensive evaluation of a city's human capital, encompassing scale expansion and structural optimization. This approach allows for the measurement of both the “static thickness” of human capital reserves and the “dynamic flow rate” of human capital renewal.

#### 3.2.4 Control variable

Drawing on previous studies on urban innovation, the control variables include industrial structure, digital finance, long-haul optical fiber cable density, broadband access ports, telecommunications service revenue, mobile cellular subscription penetration rate, and internet penetration rate (13, 36, 37). These city-level variables have been shown to affect urban innovation. Thus, using them as control variables provides a more accurate explanation for the effect of Health in All Policies. Table 3 provides the definitions and explanations of all variables.

**Table 3:** Definitions of variables.

**Variable types**	**Variables**	**Symbol**	**Definitions**
Dependent variable	Urban innovation	PcPatap	Per capita application patents
		PcPatau	Per capita authorization patents
Independent variable	Health in all policies index	HiAP	A composite index reflects the implementation status of “Health in All Policies”
Mediating variable	Human capital accumulation	HCA1	The ratio of the difference between the current period resident population and the previous period resident population to the current period household registered population
		HCA2	The ratio of the number of students enrolled in regular undergraduate and junior college programs to the household registered population (%)
Control variable	Industrial structure	Struc	The ratio of the GDP contribution from the Tertiary Sector to that of the Secondary Sector
	Inclusive digital finance index	Difi	A composite index reflecting the digital finance service
	Digital infrastructure	Haul	Long-haul optical fiber cable density
		Access	Per capita broadband access ports
		Tel	Per capita telecommunications service revenue
		Cellular	Mobile cellular subscription penetration rate
		Inter	Internet penetration rate

Given the unavailability of prefecture-level data on optical fiber cable route length and mobile cellular base station quantity, this study employs a proportional allocation method based on each city's share of provincial telecom service revenue to estimate municipal-level infrastructure metrics from provincial aggregate data.

(1) Industrial structure (Struc) is reflected by the ratio of the GDP contribution from the Tertiary Sector to that of the Secondary Sector, denoted by Struc.


(5) $$\begin{array}{c} Industrial\;structure\;\left(Struc\right)\\ =\frac{GDP\;contribution\;from\;the\;Tertiary\;Sector}{GDP\;contribution\;from\;the\;Secondary\;Sector} \end{array}$$

(2) Inclusive Digital Finance Index (Difi) refers to the implementation status of the digital finance service, denoted by Difi. Specifically, the Peking University Digital Inclusive Finance Index (hereinafter referred to as DIFI) provides a quantitative assessment of regional digital financial services across China, capturing both the inclusiveness and developmental progress of digital finance. The index employs a hybrid methodology combining the Analytic Hierarchy Process (AHP) with Principal Component Analysis (PCA). Its indicator system spans multiple financial service domains—including payment systems, credit services, insurance products, and funds—while systematically accounting for regional disparities in inclusive finance provision.

(3) Long-haul Optical Fiber Cable Density (Haul) is reflected by the ratio of route length of long-haul Optical Fiber Cables to the area of administrative divisions, denoted by Haul.


(6) $$\begin{array}{c} Long-haul\;Optical\;Fiber\;Cable\;Density\;\left(Haul\right)\\ =\frac{Route\;Length\;of\;Long-haul\;Optical\;Fiber\;Cables}{Area\;of\;Administrative\;Divisions} \end{array}$$

(4) Per Capita Broadband Access Ports (Access) is reflected by the ratio of total broadband access ports to the total population, denoted by Access.


(7) $$\begin{array}{c} Per\;Capita\;Broadband\;Access\;Ports\;\left(Access\right)\\ =\frac{Total\;Broadband\;Access\;Ports}{Total\;Population} \end{array}$$

(5) Per Capita Telecommunications Service Revenue (Tel) is reflected by the ratio of total telecommunications service revenue to the total population, denoted by Tel.


(8) $$\begin{array}{c} Per\;Capita\;Telecommunications\;Service\;Revenue\;\left(Tel\right)=\\ \frac{Total\;Telecommunications\;Service\;Revenue}{Total\;Population} \end{array}$$

(6) Mobile Cellular Subscription Penetration Rate (Cellular) is reflected by the ratio of mobile cellular subscribers to the total population, denoted by Cellular.


(9) $$\begin{array}{c} Mobile\;Cellular\;Subscription\;Penetration\;Rate\;\left(Cellular\right)=\\ \frac{Mobile\;Cellular\;Subscribers}{Total\;Population} \end{array}$$

(7) Internet Penetration Rate (Inter) is reflected by the ratio of broadband internet subscriptions to the total population, denoted by Inter.


(10) $$\begin{array}{c} Internet\;Penetration\;Rate\;\left(Inter\right)=\frac{Broadband\;Internet\;Subscriptions}{Total\;Population} \end{array}$$

In Equations 6–10, given data availability constraints at prefecture-level cities, this study selects four control variables across two dimensions of digital infrastructure development—inputs and outputs. The selected indicators include: long-haul optical fiber cable density, per capita broadband access ports, per capita telecommunications service revenue, mobile cellular subscription penetration rate, and internet penetration rate.

### 3.3 Empirical model

#### 3.3.1 Two-way fixed-effects model

To test the relationship between Health in All Policies (HiAP) and urban innovation in H1a, we take the number of application patents per capita (PcPatap) as the dependent variable, the HiAP index as the independent variable, and add control variables to establish the following equation:


(11) $$\begin{array}{c} PcPatap_{it}=\beta_{0}+\beta_{1}^{*}HiAP_{it}+\beta_{2}^{*}Struc_{it}+\beta_{3}^{*}Difi_{it}+\beta_{4}^{*}Haul_{it}\\ +\beta_{5}^{*}Access_{it}+\beta_{6}^{*}Tel_{it}+\beta_{7}^{*}Cellular_{it}+\beta_{8}^{*}Inter_{it}+\varepsilon_{it} \end{array}$$

To test the relationship between Health in All Policies (HiAP) and urban innovation in H1b, we take the number of authorization patents per capita (PcPatau) as the dependent variable, HiAP index as the independent variable, and add control variables to establish the following equation:


(12) $$\begin{array}{c} {PcPatau}_{it}=\beta_{0}+\beta_{1}^{*}HiAP_{it}+\beta_{2}^{*}Struc_{it}+\beta_{3}^{*}Difi_{it}+\beta_{4}^{*}Haul_{it}\\ +\beta_{5}^{*}Access_{it}+\beta_{6}^{*}Tel_{it}+\beta_{7}^{*}Cellular_{it}+\beta_{8}^{*}Inter_{it}+\varepsilon_{it} \end{array}$$

In Equations 11, 12, i and t represent province and year. β_0_ is the constant term, β_1_ to β_8_ are the regression estimation coefficients of each variable. PcPatap_it_ and PcPatau_it_, respectively represent application patents and authorization patents, reflecting the knowledge innovation and product innovation of a city in China. HiAP_it_ represents the level of Health in All Policies, which is the independent variable of this study. Control variables include industrial structure, digital finance, long-haul optical fiber cable density, broadband access ports, telecommunications service revenue, mobile cellular subscription penetration rate, and internet penetration rate. ε_it_ is the random error term.

#### 3.3.2 Mediation effects model

To test the mediating effect, we use the stepwise regression analysis method. First, we conduct the regression between HiAP and urban innovation to determine whether the coefficient of HiAP is significant. Second, the regressions of HiAP and population mobility (HCA1), and HiAP and talent stock (HCA2) are conducted to observe whether the coefficient of HiAP is significant. In Equations 13, 14, we conduct the regression of urban innovation, population mobility, and HiAP. In Equations 15, 16, we also conduct the regression of urban innovation, talent stock, and HiAP.

To test H3a, we take the urban innovation as the dependent variable, add HiAP, population mobility (HCA1), and control variables to construct the following regression equation:


(13) $$\begin{array}{c} PcPatap_{it}=\beta_{0}+\beta_{1}^{*}HiAP_{it}+\;\beta_{2}^{*}{HCA1}_{it}\;+\;\beta_{3}^{*}Struc_{it}\\ +\beta_{4}^{*}Difi_{it}+\beta_{5}^{*}Haul_{it}+\beta_{6}^{*}Access_{it}+\beta_{7}^{*}Tel_{it}+\beta_{8}^{*}Cellular_{it}\\ +\beta_{9}^{*}Inter_{it}+\varepsilon_{it} \end{array}$$

(14) $$\begin{array}{c} PcPatau_{it}=\beta_{0}+\beta_{1}^{*}HiAP_{it}\;+\;\beta_{2}^{*}{HCA1}_{it}\;+\;\beta_{3}^{*}Struc_{it}\;\\ +\;\beta_{4}^{*}Difi_{it}\;+\;\beta_{5}^{*}Haul_{it}\;+\;\beta_{6}^{*}Access_{it}\;+\;\beta_{7}^{*}Tel_{it}\;+\\ +\;\beta_{8}^{*}Cellular_{it}\;\beta_{9}^{*}Inter_{it}\;+\;\varepsilon_{it} \end{array}$$

To test H3b, we take the urban innovation as the dependent variable, add HiAP, talent stock (HCA2), and control variables to construct the following regression equation:


(15) $$\begin{array}{c} PcPatap_{it}=\beta_{0}+\beta_{1}^{*}HiAP_{it}+\;\beta_{2}^{*}{HCA2}_{it}\;+\;\beta_{3}^{*}Struc_{it}+\beta_{4}^{*}Difi_{it}\\ +\beta_{5}^{*}Haul_{it}+\beta_{6}^{*}{Access}_{it}+\beta_{7}^{*}Tel_{it}+\beta_{8}^{*}Cellular_{it}+\beta_{9}^{*}Inter_{it}+\varepsilon_{it} \end{array}$$

(16) $$\begin{array}{c} PcPatau_{it}=\beta_{0}\;+\;\beta_{1}^{*}HiAP_{it}\;+\;\beta_{2}^{*}{HCA2}_{it}\;+\;\beta_{3}^{*}Struct_{it}\;+\\ \;\beta_{4}^{*}Difi_{it}\;+\;\beta_{5}^{*}Haul_{it}\;+\;\beta_{6}^{*}Access_{it}\;+\;\beta_{7}^{*}Tel_{it}\;+\;\beta_{8}^{*}Cellular_{it}\;+\\ +\;\beta_{9}^{*}Inter_{it}\;+\;\varepsilon_{it} \end{array}$$

In Equations 13–16, i and t represent province and year. β_0_ is the constant term, β_1_ to β_8_ are the regression estimation coefficients of each variable. PcPatap_it_ and PcPatau_it_, respectively represent application patents and authorization patents, reflecting the knowledge innovation and product innovation of urban innovation. HCA1 represents the population mobility, and HCA2 represents the talent stock, reflecting the human capital accumulation. HiAP_it_ represents the level of Health in All Policies, which is the independent variable of this study. Control variables include industrial structure, digital finance, long-haul optical fiber cable density, broadband access ports, telecommunications service revenue, mobile cellular subscription penetration rate, and internet penetration rate. ε_it_ is the random error term.

### 3.4 Method implementation

All analyses were performed using Stata/MP 16.0. The two-way fixed effects regressions utilized the “reghdfe” package (38, 39) to address high-dimensional fixed effects. For the mediation effect analysis, we employed the sgmediation 2 package to perform the Sobel Z-test. To further reinforce the robustness of the mediation results, a bootstrap test (with 1,000 replications) was also conducted. Furthermore, to verify the parallel trend assumption for the Difference-in-Differences (DID) model, we utilized the coefplot package to visualize the event-study plot, which graphically demonstrates that no statistically significant differences in trends existed between the treatment and control groups prior to the policy intervention.

### 3.5 Statistical descriptions

The dependent variable is urban innovation. The mean value, standard deviation, minimum value, and maximum value of the number of application patents are respectively 11,552.70, 26,672.10, 41.00, and 320,813.00; The above four contents of the number of authorization patents are 8,145.73, 19,102.32, 33.00, and 279,533.00, respectively. As for the independent variable, the implementation status of Health in All Policies in each city, its mean value is 21.12, standard deviation is 4.19, minimum value is 13.16, and maximum value is 48.01. Obviously, there are differences in urban innovation and the implementation status of Health in All Policies in cities at the prefecture level. Table 4 presents the summary statistics.

**Table 4:** Summary statistics.

**Variable types**	**Variables**	**Obs**	**Mean**	**SD**	**Min**	**Max**
Dependent variable	PcPatap	2,414	21.71	43.08	0.17	511.52
	PcPatau	2,414	15.45	31.53	0.12	443.00
Independent variable	HiAP	2,245	21.12	4.19	13.16	48.01
Mediating variable	HCA1	1,860	0.01	0.03	−0.30	0.40
	HCA2	1,333	2.12	2.54	0.10	13.98
Control variable	Struc	2,147	1.11	0.60	0.00	5.35
	Difi	2,414	210.54	53.91	93.67	359.68
	Haul	2,414	0.21	0.789	0.00	22.11
	Access	2,414	0.33	0.84	0.01	22.72
	Tel	2,414	1,216.60	2,038.33	48.61	31,813.24
	Cellular	2,414	1.15	0.77	0.11	10.17
	Inter	2,414	2,909.77	2,126.76	34.72	18,901.94

## 4 Empirical results

### 4.1 Baseline results

This study employs a two-way fixed effects model based on Equation 1, to systematically assess the influence of Health in All Policies (HiAP) on urban innovation development. Table 5 presents the empirical analysis, where models (1)–(3) investigate the impact on the number of patent applications per capita (PcPatap) with three specifications: without fixed effects, with time fixed effects exclusively, and with both time and city fixed effects. Likewise, models (4)–(6) examine the effect on the number of patent authorizations per capita (PcPatau) as the dependent variable.

**Table 5:** Primary regression results.

**Variables**	**(1)**	**(2)**	**(3)**	**(4)**	**(5)**	**(6)**
	**PcPatap**	**PcPatap**	**PcPatap**	**PcPatau**	**PcPatau**	**PcPatau**
HiAP	8.052^***^	6.788^***^	6.022^***^	5.951^***^	5.101^***^	4.604^***^
	(1.200)	(1.746)	(1.282)	(0.973)	(1.422)	(0.974)
Struc	−6.392	−5.113	−3.576	−6.247^*^	−5.050	−3.383
	(4.243)	(4.947)	(4.197)	(3.326)	(3.838)	(3.751)
Difi	−0.094^***^	0.267	0.382^***^	−0.052^***^	0.184	0.299^***^
	(0.021)	(0.158)	(0.092)	(0.016)	(0.128)	(0.074)
Haul	−0.644	−0.694	−1.864^*^	−0.410	−0.124	−0.654^*^
	(1.093)	(1.179)	(1.063)	(0.666)	(0.641)	(0.358)
Access	−1.682^**^	−0.237	1.893^**^	0.103	−0.544	0.832^*^
	(0.761)	(0.962)	(0.865)	(0.748)	(0.755)	(0.407)
Tel	0.001^**^	0.000	−0.001^**^	0.000	0.000	−0.001^**^
	(0.000)	(0.000)	(0.000)	(0.000)	(0.000)	(0.000)
Cellular	−1.759	−1.574	−10.376^***^	−1.132	−1.193	−8.052^***^
	(1.916)	(1.583)	(2.123)	(1.341)	(1.203)	(1.754)
Inter	0.001	0.000	0.000	0.000	0.000	−0.000
	(0.001)	(0.001)	(0.001)	(0.001)	(0.001)	(0.001)
_cons	−122.272^***^	−172.750^***^	−169.959^***^	−92.788^***^	−125.001^***^	−130.790^***^
	(18.750)	(20.422)	(35.558)	(15.441)	(15.422)	(23.828)
Year FE	NO	YES	YES	NO	YES	YES
City FE	NO	NO	YES	NO	NO	YES
R^2^	0.668	0.689	0.916	0.643	0.658	0.891
*N*	1,995	1,995	1,992	1,995	1,995	1,992

Standard errors in parentheses. ^*^p < 0.10, ^**^p < 0.05, ^***^p < 0.01. Sample restricted to complete-case observations. Singletons excluded per Correia (70).

The empirical results indicate a significant positive impact of the Health in All Policies (HiAP) on urban innovation outputs, increasing both patent applications per capita and patent authorizations per capita. This finding remains consistent across all three model specifications. Specifically, model (3) indicates that under time-city two-way fixed effects, a one-unit rise in the HiAP index significantly leads to a substantial increase in patent applications per capita (coefficient = 6.022, *p* < 0.01). Likewise, model (6) shows that under two-way fixed effects, a one-unit increase in the HiAP index significantly increases patent authorizations per capita (coefficient = 4.604, *p* < 0.01). These results align closely with theoretical expectations, indicating that the integration of health considerations into urban governance policies significantly enhances innovation capacity at the city level. Furthermore, the observed difference in coefficients between patent applications and authorizations, with the former being notably higher, supports the notion of a filtering mechanism in the patent review process, suggesting that not all applications meet quality standards. This discovery offers micro-level support for the implementation of the “Healthy China” strategy, highlighting that the deep integration of health policies into urban governance systems can stimulate knowledge spillovers and human capital accumulation, thereby fostering the establishment of a high-quality development framework.

### 4.2 Robustness tests

#### 4.2.1 Difference in difference model

**Figure 2:**
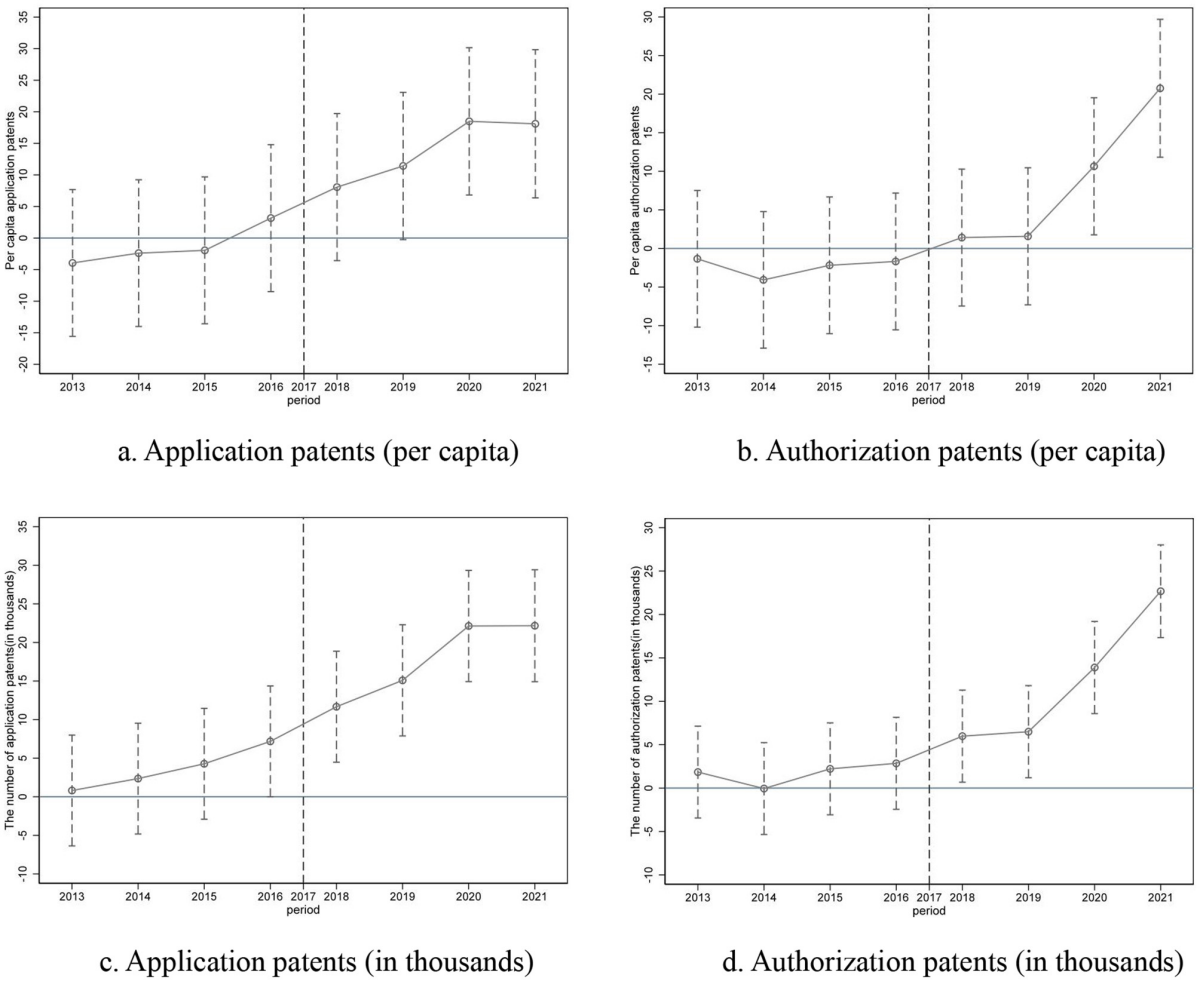
Parallel trends test. **(a)** Application patents (per capita). **(b)** Authorization patents (per capita). **(c)** Application patents (in thousands). **(d)** Authorization patents (in thousands).

To ensure the robustness of the findings, this study employs a difference-in-differences (DID) methodology, substituting the independent variable with the China Healthy Cities Policy Pilot. In 2016, China designated 38 cities as pilot cities for the healthy cities policy (30). This study uses the interaction term between the pilot city dummy variable and the policy timing as a proxy for the independent variable. As demonstrated in Table 6, the healthy cities policy pilot significantly boosts both the number of patent applications per capita (PcPatap; coefficient = 15.643, *p* < 0.01) and the number of patent authorizations per capita (PcPatau; coefficient = 11.136, *p* < 0.01), confirming the positive impact of health policy shocks on innovation outputs. Regarding the parallel trends test for the DID model in Robustness Check 1, we conducted separate tests for two types of dependent variables and present their respective dynamic effect diagrams, demonstrating robust results (Figure 2).

**Table 6:** Robustness tests results of DID model.

	**(1)**	**(2)**	**(3)**	**(4)**
**Variables**	**PcPatap**	**PcPatau**	**Patap (in thousands)**	**Patau (in thousands)**
DID	15.643^***^	11.136^***^	13.801^***^	10.388^***^
	(3.299)	(2.205)	(4.209)	(3.047)
Control variables	YES	YES	YES	YES
Year FE	YES	YES	YES	YES
City FE	YES	YES	YES	YES
R^2^	0.912	0.887	0.878	0.836
N	2,146	2,146	2,146	2,146

Standard errors in parentheses. ^*^p < 0.10, ^**^p < 0.05, ^***^p < 0.01. Sample restricted to complete-case observations. Singletons excluded per Correia (70).

It is important to highlight that the baseline model favors the HiAP index over the policy pilot for two main reasons. Firstly, the healthy cities policy pilot is a relatively broad concept without clear action standards or measures, and non-pilot cities may also undertake spontaneous health policy innovations. Secondly, the HiAP index, derived through the entropy-weighted Technique for Order of Preference by Similarity to Ideal Solution (TOPSIS) method, offers a comprehensive assessment of policy coherence across five dimensions: economic support, public services, environmental governance, cultural dissemination, and medical infrastructure. This approach allows for a more precise evaluation of the impacts of Health in All Policies (HiAP) across various dimensions, whereas a binary policy variable may struggle to capture such multidimensional dynamics.

#### 4.2.2 Replace the dependent variable

We use the China City and Industrial Innovation (CCIC) Index as an alternative dependent variable. The CCIC Index, adjusted for patent value, calculates the average value of patents by age and aggregates the value of patents across different ages. Similar to physical capital stock, it quantifies intangible capital stock based on patent value (40). As indicated in Table 7, model (1), a one-unit increase in the HiAP index leads to a significant 30.448-unit rise in the CCIC Index (*p* < 0.05). This result validates the robustness of the baseline findings from the perspective of patent value stock.

**Table 7:** Other robustness tests results.

**Variables**	**(1)**	**(2)**	**(3)**	**(4)**	**(5)**	**(6)**	**(7)**
	**CIIC**	**PcPatap_growth**	**PcPatau_growth**	**F.PcPatap**	**F.PcPatau**	**Patap (in thousands)**	**Patau (in thousands)**
HiAP	30.448^**^	0.037	1.461^**^	5.985^***^	5.158^***^	4.775^***^	3.748^***^
	(14.441)	(0.184)	(0.565)	(1.231)	(1.088)	(1.270)	(0.958)
Control variables	YES	YES	YES	YES	YES	YES	YES
Year FE	YES	YES	YES	YES	YES	YES	YES
City FE	YES	YES	YES	YES	YES	YES	YES
R^2^	0.835	0.800	0.444	0.934	0.905	0.912	0.870
*N*	2,146	1,740	1,740	1,739	1,739	1,992	1,992

Standard errors in parentheses. ^*^p < 0.10, ^**^p < 0.05, ^***^p < 0.01. Sample restricted to complete-case observations. Singletons excluded per Correia (70).

#### 4.2.3 Incremental model of the dependent variable

We construct an incremental model, utilizing the year-on-year growth in PcPatap and PcPatau as substitute dependent variables. This approach overcomes the static limitations of traditional stock indicators, enabling a more sensitive capture of the dynamic evolution of innovation capacity. In Table 7, models (2)–(3) demonstrate the effect of the HiAP index on patent growth. The results show that an increase in the HiAP index tends to promote the growth in PcPatap, though without statistical significance, while a one-unit increase in the HiAP index significantly boosts the growth in PcPatau (coefficient = 1.461, *p* < 0.05). Compared to the number of patent authorizations, the number of patent applications has a higher probability of containing low-quality innovations. The noise from these non-viable patents dilutes the significance. In other words, the non-significance reflects this measurement attenuation, not effect absence. It is evident that the impact of Health in All Policies (HiAP) on innovation is to enhance the quality of innovation (authorizations), not just the quantity of innovation (applications). Given that Patau as product innovation better reflects substantive technological breakthroughs and market value, the significant increase in Patau growth supports the reliability of the findings.

#### 4.2.4 Lagged effects of health index on innovation

Considering the time-lagged effects of health policies on human capital accumulation, this study constructs a lagged effects model, lagging the dependent variable (F.PcPatap and F.PcPatau) by one period, to assess the influence on patent outputs in the subsequent year. As shown in Table 7, models (4)–(5), under the two-way fixed effects model, a one-unit increase in the HiAP index significantly increases the next year's PcPatap (coefficient = 5.985, *p* < 0.01) and PcPatau (coefficient = 5.158, *p* < 0.01). This finding not only confirms the persistence of policy effects but also effectively mitigates endogeneity concerns arising from reverse causality.

#### 4.2.5 Lagged effects of health index on innovation

To delve deeper into the robustness test, we use the Patap (in thousands) and Patau (in thousands) as alternative dependent variables. As indicated in Table 7, models (6)–(7), a one-unit increase in the HiAP index leads to a significant 4.775-unit rise in Patap (in thousands) and a significant 3.748-unit rise in Patap (in thousands; *p* < 0.01). This result validates the robustness of the baseline findings again.

#### 4.2.6 Sensitivity analyses with alternative weighting schemes

The independent variable HiAP index in this paper is weighted using the Delphi method. To further ensure the robustness of the empirical results, we re-allocated the weights of the independent variable using the equal weighting method. The results show that HiAP still robustly promotes innovation output (PcPatap: β = 9.391, *p* < 0.01, PcPatau: β = 7.053, *p* < 0.01).

### 4.3 Sub-dimensional effects

While the aggregated HiAP index demonstrates a robust association with innovation (Tables 5–8), the mechanisms driving this relationship may vary significantly across policy domains. To disentangle these dynamics, we estimate separate models for each sub-dimension of HiAP policies: health in economy (Panel A), health in public service (Panel B), health in environment (Panel C), health in culture (Panel D), and health in medical care (Panel E).

**Table 8:** Sensitivity analyses with equal weighting schemes.

**Variables**	**(1)**	**(2)**
	**PcPatap**	**PcPatau**
HiAP_Equal Weighting	9.391^***^ (3.051)	7.053^***^ (2.411)
Struc	−4.133 (4.273)	−3.808 (3.789)
Difi	0.456^***^ (0.118)	0.357^***^ (0.092)
Haul	−1.808 (1.062)	−0.609 (0.377)
Access	2.093^**^ (0.850)	0.982^**^ (0.409)
Tel	−0.001^**^ (0.000)	−0.001^**^ (0.000)
Cellular	−11.796^***^ (2.891)	−9.159^***^ (2.319)
Inter	0.000 (0.001)	0.000 (0.001)
_cons	−142.920^***^ (35.356)	−109.121^***^ (23.706)
Year FE	YES	YES
City FE	YES	YES
R^2^	0.908	0.884
*N*	1,992	1,992

Standard errors in parentheses. ^*^p < 0.10, ^**^p < 0.05, ^***^p < 0.01. Sample restricted to complete-case observations. Singletons excluded per Correia (70).

Table 9 reveals differences in both effect magnitudes and statistical significance, underscoring the need for policymakers to prioritize specific strategies rather than relying on uniform interventions.

**Table 9:** Sub-dimensional effects of HiAP index.

**Variables**	**(1)**	**(2)**
	**PcPatap**	**PcPatau**
**Panel A: health in economy**	4.259^***^ (0.553)	3.638^***^ (0.558)
Control variables	YES	YES
Year FE	YES	YES
City FE	YES	YES
R^2^	0.921	0.902
*N*	2,031	2,031
**Panel B: health in public service**	0.198 (2.227)	0.216 (1.851)
Control variables	YES	YES
Year FE	YES	YES
City FE	YES	YES
R^2^	0.908	0.883
*N*	2,138	2,138
**Panel C: health in environment**	−0.061 (0.262)	−0.066 (0.248)
Control variables	YES	YES
Year FE	YES	YES
City FE	YES	YES
R^2^	0.909	0.884
N	2,130	2,130
**Panel D: health in culture**	10.640^***^ (1.123)	6.394^***^ (1.136)
Control variables	YES	YES
Year FE	YES	YES
City FE	YES	YES
R^2^	0.928	0.896
N	2,131	2,131
**Panel E: health in medical care**	1.349^**^ (0.546)	0.810^*^ (0.441)
Control variables	YES	YES
Year FE	YES	YES
City FE	YES	YES
R^2^	0.910	0.885
N	2,146	2,146

Standard errors in parentheses. ^*^p < 0.10, ^**^p < 0.05, ^***^p < 0.01. Sample restricted to complete-case observations. Singletons excluded per Correia (70).

Health policies embedded in economic systems (Panel A: β = 4.259, *p* < 0.01, β = 3.638, *p* < 0.01) and cultural frameworks (Panel D: β = 10.640, *p* < 0.01, β = 6.394, *p* < 0.01) exert the strongest positive effects on innovation outputs (PcPatap and PcPatau). These findings align with endogenous growth theory, where economic capital accumulation and cultural creativity synergize to stimulate R&D activities. Health in the economy enhances labor productivity and attracts industrial investment (41, 42), thereby driving innovation outputs. In contrast, health in culture exerts a disproportionately stronger effect on innovation compared to other dimensions. This is because health-oriented cultural initiatives strengthen social capital, foster knowledge sharing and cross-sector collaboration (43, 44) , and populations with higher health literacy are more receptive to adopting new technologies (45).

The coefficients for Health in Public Service (Panel B) and Health in Environment (Panel C) are not statistically significant, suggesting that these domains may operate through longer-term channels (e.g., population health improvements) rather than directly spurring immediate innovation.

Health in medical care (Panel E) exhibits marginally significant positive impact (PcPatap: β = 1.349, *p* < 0.05, PcPatau: β = 0.810, *p* < 0.10), implying that healthcare infrastructure and accessibility are fundamental to urban innovation. Although its direct impact is weaker than economic or cultural dimensions, healthcare plays an enabling role in sustaining long-term innovation ecosystems. Improved healthcare accessibility reduces workforce morbidity and extends the productive lifespan of high-skilled workers (46, 47). For example, chronic disease management programs targeting engineers or scientists directly prevent early retirements due to health shocks, thereby preserving institutional knowledge and R&D continuity (48).

### 4.4 Heterogeneity analysis

This study systematically examines the heterogeneous innovation effects of the HiAP across four dimensions, including administrative hierarchy, regional location, geographical demarcation, and city size (Table 10). These dimensions were selected to reveal how policy effectiveness is moderated by structural disparities in urban endowments. First, the administrative hierarchy (provincial capitals vs. non-capitals) reflects institutional disparities in resource allocation and policy implementation capacity (49). Second, regional location (eastern, central, and western city distribution) and the north-south demarcation reflect varying degrees of marketization and industrial structure (50). Finally, city size (megacities/super-large cities vs. small/medium cities) highlights the amplifying or diminishing effects of agglomeration economies on policy dividends (51).

**Table 10:** Heterogeneity analysis results.

**Variables**	**(1)**	**(2)**	**(3)**	**(4)**	**(5)**	**(6)**	**(7)**	**(8)**
	**PcPatap**	**PcPatau**	**PcPatap**	**PcPatau**	**PcPatap**	**PcPatau**	**PcPatap**	**PcPatau**
HiAP	4.650^***^	3.513^***^	8.418^***^	6.682^***^	5.110^***^	3.759^***^	3.361^***^	2.296^***^
	(1.339)	(0.991)	(1.390)	(1.159)	(1.148)	(0.863)	(0.878)	(0.792)
**Provincial capital#c.HiAP (comparison: not provincial capital)**								
provincial capital#HiAP	3.009^***^	2.395^***^						
	(0.636)	(0.398)						
**East, central, and west city distribution#c.HiAP (comparison: east)**								
central#HiAP			−4.036^***^	−3.634^***^				
			(1.095)	(0.999)				
west#HiAP			−4.620^***^	−3.935^***^				
			(0.966)	(0.893)				
**North and south city distribution #HiAP (comparison: north)**								
south#HiAP					1.496	1.387		
					(1.050)	(0.942)		
**Super-large cities and Megacities#HiAP (Comparison: not Super-large cities or Megacities)**								
city#HiAP							5.774^***^	5.011^***^
							(1.448)	(1.502)
Control Var	YES	YES	YES	YES	YES	YES	YES	YES
Year FE	YES	YES	YES	YES	YES	YES	YES	YES
City FE	YES	YES	YES	YES	YES	YES	YES	YES
R^2^	0.919	0.895	0.927	0.906	0.917	0.893	0.926	0.905
*N*	1,992	1,992	1,992	1,992	1,992	1,992	1,992	1,992

Standard errors in parentheses. ^*^p < 0.10, ^**^p < 0.05, ^***^p < 0.01. Sample restricted to complete-case observations. Singletons excluded per Correia (70).

In the context of administrative hierarchy, the impact of policies in provincial capitals (interaction term PcPatap: β = 4.650, *p* < 0.01, PcPatau: β = 3.513, *p* < 0.01) exceeds that of non-capital cities. It underscores the resource siphoning effect of provincial capitals as regional power centers (e.g., special fiscal allocations and concentration of high-tier medical institutions) in amplifying health policy implementation (52).

Moreover, regional disparities reveal a nuanced gradient: the innovation-promoting effect of HiAP is strongest in eastern China. Specifically, compared to the eastern region, both the central (PcPatap: β = −4.036, *p* < 0.01, PcPatau: β = −3.634, *p* < 0.01) and western regions (PcPatap: β = −4.620, *p* < 0.01, PcPatau: β = −3.935, *p* < 0.01) show negative interactions. This pattern likely stems from dual challenges in central and western regions. Constrained fiscal capacity forces local governments to trade off health expenditures against infrastructure investments, creating resource crowding-out effects. While bureaucratic inflexibility leads to one-size-fits-all policy implementation that mismatches local innovation ecosystems (49).

In contrast, the north-south demarcation shows weak heterogeneity, no statistically significance. This is because China's centralized health governance system promotes policy standardization. The high degree of uniformity in policy objectives and the rigidity of assessment in health policies significantly weaken the differences between the southern and northern regions. Also, unlike climate policies—which exhibit latitude-dependent sensitivity to thermal extremes and precipitation—HiAP demonstrates low environmental determinism. Policies with analogous low geoclimatic sensitivity similarly show low north-south divergence (53, 54).

Notably, city size heterogeneity reveals the disruptive impact of agglomeration economies. The innovation returns from HiAP in megacities and super-large cities are significantly greater than in small and medium cities (PcPatap: β = 5.774, *p* < 0.01, PcPatau: β = 5.011, *p* < 0.01). This disparity can be attributed to three advantages of megacities—high-density talent pools and industrial chains accelerate health technology commercialization, diversified financing systems alleviate capital constraints, and institutional flexibility enables policy experimentation to unlock dividends (Table 10).

## 5 Mediating effect analysis

We use the stepwise regression analysis method to explore the mechanisms by which Health in All Policies (HiAP) impacts urban innovation. Human capital accumulation serves as the mediating variable and is measured through two indicators: population mobility (HCA1) and talent stock (HCA2). As shown in Table 11, the regression coefficient of HiAP on PcPatap and PcPatau is separately 6.02 and 4.60 in column (1) and (2), which is significant at the 1% level. In column (3), the coefficient of HiAP is significant at the 5% level. In column (4) and (5), the coefficients of HiAP and HCA1 are significant, which can be considered a mediating effect. In column (6), the coefficient of HiAP is significant at the 5% level. In column (7) and (8), the coefficients of HiAP and HCA2 are significant, which can be considered a mediating effect.

**Table 11:** Mediating analysis of human capital accumulation.

**Variables**	**(1)**	**(2)**	**(3)**	**(4)**	**(5)**	**(6)**	**(7)**	**(8)**
	**PcPatap**	**PcPatau**	**HCA1**	**PcPatap**	**PcPatau**	**HCA2**	**PcPatap**	**PcPatau**
HiAP	6.022^***^	4.604^***^	0.005^**^	5.654^***^	6.010^***^	0.072^**^	1.990^*^	4.073^**^
	(1.282)	(0.974)	(0.002)	(1.215)	(1.100)	(0.031)	(0.977)	(1.584)
HCA1				58.257^**^	50.295^**^			
				(22.573)	(23.132)			
HCA2							2.696^**^	2.910^***^
							(1.196)	(0.905)
Struc	−3.576	−3.383	0.002	−5.039	−7.561^*^	0.077	0.099	−0.906
	(4.197)	(3.751)	(0.004)	(4.736)	(3.868)	(0.082)	(2.061)	(1.829)
Difi	0.382^***^	0.299^***^	0.000	0.425^***^	−0.021	0.001	0.094	−0.068
	(0.092)	(0.074)	(0.000)	(0.124)	(0.019)	(0.003)	(0.097)	(0.184)
Haul	−1.864^*^	−0.654^*^	−0.002	−1.358	−0.225	−0.017	−0.434	0.306
	(1.063)	(0.358)	(0.003)	(0.970)	(0.701)	(0.018)	(0.435)	(0.668)
Access	1.893^**^	0.832^*^	−0.000	1.578	−0.168	0.018	0.370	−0.209
	(0.865)	(0.407)	(0.003)	(0.941)	(0.959)	(0.015)	(0.295)	(0.523)
Tel	−0.001^**^	−0.001^**^	0.000	−0.001^**^	0.000	−0.000^**^	−0.000	−0.000
	(0.000)	(0.000)	(0.000)	(0.000)	(0.000)	(0.000)	(0.000)	(0.000)
Cellular	−10.376^***^	−8.052^***^	−0.004	−8.844^***^	−0.039	−0.082	−3.895	−9.700^**^
	(2.123)	(1.754)	(0.008)	(2.570)	(1.341)	(0.103)	(3.131)	(3.888)
Inter	0.000	−0.000	0.000^**^	−0.000	0.000	−0.000	0.000	0.001
	(0.001)	(0.001)	(0.000)	(0.001)	(0.001)	(0.000)	(0.000)	(0.001)
_cons	−169.959^***^	−130.790^***^	−0.172^**^	−176.765^***^	−100.508^***^	0.306	−44.042	−50.608
	(35.558)	(23.828)	(0.064)	(40.860)	(17.185)	(1.007)	(37.891)	(47.312)
Year FE	YES	YES	YES	YES	YES	YES	YES	YES
City FE	YES	YES	YES	YES	YES	YES	YES	YES
Sobel Z				0.381^***^	0.174^**^		−0.482^*^	−0.642^**^
R^2^	0.916	0.891	0.355	0.938	0.665	0.992	0.986	0.962
N	1,992	1,992	1,520	1,520	1,533	991	991	991

Standard errors in parentheses. ^*^p < 0.10, ^**^p < 0.05, ^***^p < 0.01. Sample restricted to complete-case observations. Singletons excluded per Correia (70).

These outcomes provide empirical support for Hypotheses H3a and H3b, indicating that HiAP promotes human capital accumulation through improved health-related social determinants, such as enhanced healthcare accessibility and sustainable urban environments. These improvements attract skilled labor and contribute to better educational outcomes [(73); 42]. Previous research has shown that human capital accumulation plays a crucial role in fostering innovation, facilitated by scale agglomeration and knowledge spillover effects (55, 56). This study reveals that human capital accumulation serves as a mediator in the relationship between HiAP and innovation. This mediation signifies that dynamic labor inflows enhance urban innovation by boosting the labor supply and facilitating knowledge transfer (57).

To delve deeper into the mediation pathways, the percentile bootstrap test step of deviation correction is used to test the intermediary effect. The indirect effect of HiAP on urban innovation (the number of application patents per capita) through population mobility is significant [index = 0.381, 95% CI (0.089, 0.673)], while the indirect effect of HiAP on urban innovation (the number of authorization patents per capita) through population mobility is not significant. In addition, Table 12 shows that the confidence interval does not include 0, indicating that the indirect impact of HiAP on urban innovation through talent stock [index = −0.482, 95 per cent CI (−0.904, −0.060); index = −0.642, 95 per cent CI (−1.001, −0.282)] is significant, hypothesis 3b is verified again.

**Table 12:** Bootstrap results for the mediation effect.

**Mediating variables**		**Observed Coef**.	**Std. Err**	***z***	**95% CI**
HCA1-PcPatap	Indirect effect	0.381	0.149	2.56	(0.089, 0.673)
	Direct effect	8.104	0.488	16.62	(7.148, 9.060)
	Total effect	8.485	0.441	19.24	(7.620, 9.349)
HCA1-PcPatau	Indirect effect	0.174	0.099	1.77	(−0.019, 0.367)
	Direct effect	6.010	0.438	13.72	(5.152, 6.686)
	Total effect	6.184	0.394	15.71	(5.413, 6.955)
HCA2-PcPatap	Indirect effect	−0.482	0.215	−2.24	(−0.904, −0.060)
	Direct effect	9.556	0.797	12.00	(7.995, 11.118)
	Total effect	9.0746	0.686	13.23	(7.730, 10.419)
HCA2-PcPatau	Indirect effect	−0.642	0.183	−3.50	(−1.001, −0.282)
	Direct effect	7.187	0.714	10.07	(5.788, 8.586)
	Total effect	6.545	0.611	10.71	(5.347, 7.744)

Standard errors in parentheses. ^*^p < 0.10, ^**^p < 0.05, ^***^p < 0.01. Sample restricted to complete-case observations. Singletons excluded per Correia (70).

## 6 Discussions and conclusions

Drawing on migration theory, innovation ecosystem theory, and the Chinese institutional context, this study investigates the relationship among Health in All Policies (HiAP), human capital accumulation, and urban innovation across 271 prefecture-level and above cities from 2013 to 2021. The empirical analysis reveals a significant positive association between HiAP and urban innovation. Furthermore, human capital accumulation plays a mediating role in the relationship between HiAP and urban innovation. Population mobility (HCA1) and talent stock (HCA2) are identified as mediators. The relationship between HiAP and urban innovation varies across cities based on population size, administrative level, and geographical location. These findings deepen our understanding of HiAP's role in shaping urban innovation and provide valuable insights for policymakers and enterprises to strengthen cities' innovation capacity in pursuit of sustainable development, thereby generating economic, social, and environmental benefits through coordinated innovation efforts.

This study finds that Health in All Policies (HiAP) is positively associated with urban innovation, extending policy innovation effect research from the perspective of health (58–61). Furthermore, building on the observed robustness of the HiAP-urban innovation nexus, the model's broader relevance can be contextualized through comparative analysis with international frameworks and potential applications in diverse urbanization contexts. Conceptually, while the World Health Organization's Health in All Policies (HiAP) framework emphasizes cross-sectoral integration, our framework extends beyond interdepartmental coordination to encompass five distinct governance dimensions—economic, social, environmental, cultural, and medical—with heightened emphasis on explicit institutional embedding across these domains. Given the absence of a universal HiAP implementation model, it is imperative to examine contextually successful international practices: Ecuador's Buen Vivir national plan demonstrates measurable health outcome improvements through culturally grounded welfare restructuring, Finland's HARMONICS framework advances systemic interoperability among policy subsystems, and the Americas region offers critical examples of locally adapted HiAP initiatives (62, 63). Our integrated governance approach addresses complex urban health challenges by mainstreaming health considerations into all policy sectors—a strategy particularly salient for developing nations confronting rapid lifestyle transitions, environmental resource constraints, and mounting healthcare delivery pressures.

The relationship between Health in All Policies (HiAP) and urban innovation in China exhibits significant heterogeneity across geographical locations, city sizes, and administrative hierarchies. Such heterogeneous patterns align with findings from multiple international studies examining innovation outcomes of health-oriented public policies (64). First, principles of new economic geography indicate that innovation activities are inherently characterized by uneven spatial distributions globally, typically concentrating in core regions due to agglomeration economies and knowledge spillovers (65, 66). In developing economies specifically, empirical evidence demonstrates high spatial concentration of technological innovation capacity within China's five major urban agglomerations, while Turkey exhibits pronounced geographical clustering of innovation activities, particularly in metropolitan areas (67, 68). In addition, variations across administrative hierarchies reflect broader governance literature highlighting the comparative advantages of provincial capitals in governance effectiveness and policy implementation—factors that facilitate policy goal attainment and innovation advancement (69). Collectively, these findings demonstrate that the observed heterogeneity is either empirically validated across diverse regions or conceptually extensible to other contexts through established theoretical frameworks.

This study may have four potential marginal contributions. First, this study advances the theoretical boundaries of health policy economics by illuminating the underappreciated role of HiAP as a catalyst for urban innovation ecosystems. Our findings broaden the investigative scope of HiAP beyond conventional public health paradigms, positioning it as a multidimensional driver of knowledge-based urban development. Second, the research empirically supports the crucial role of human capital accumulation in mediating the relationship between HiAP and urban innovation, addressing a gap in existing literature. Drawing on the Chinese context, a novel theoretical proposition is proposed, suggesting that health policy instrumentation reconstitutes urban innovation landscapes through cumulative human capability augmentation. Third, combining the flow-oriented and stock-oriented perspectives to assess human capital accumulation can comprehensively evaluate the scale expansion and structural optimization of a city's human capital, measuring the “static thickness” of human capital reserves and the “dynamic flow” of human capital renewal.

Our study has several limitations that warrant further investigation. First, the neglect of spatial spillover effects constitutes a methodological limitation. While urban innovation activities inherently exhibit geographical spillover characteristics, our analytical framework presumes spatial independence among cities. This approach omits spatial econometric techniques such as the Spatial Durbin Model, potentially inflating the direct effects of HiAP while underestimating regionally coordinated impacts. Second, the measurement of HiAP requires more thorough consideration. Typically assessed through indicator systems, surrogate measures, and a binary variable, HiAP's measurement necessitates refinement. Third, the assumption of a singular mediation pathway imposes theoretical limitations. Although our analytical framework prioritizes human capital accumulation as the mediating factor, it may inadequately address concurrent mechanisms. Mono-mediation specifications tend to oversimplify the multifaceted causal pathways through which HiAP influences urban innovation. Future studies should examine the sensitivity and robustness of results when incorporating alternative mediators. Forth, although unlike multiple imputation requiring MAR assumption, linear interpolation only assumes temporal continuity between adjacent observations—a condition satisfied for our socioeconomic indicators from statistical yearbook in China. Linear interpolation may smooth authentic variations in non-random missing contexts. Future studies should apply other imputation to verify our findings. Lastly, this study overlooks the hidden and long-term effect of urban innovation. Patent metrics inadequately capture hidden innovations, potentially underestimating HiAP's effects in institutional contexts. The quantity of patents, serving as a proxy for urban innovation, is influenced by the research and development cycle. Consequently, forthcoming research should capture dynamic temporal relationships through extensive panel analyses.

## Data Availability

Publicly available datasets were analyzed in this study. This data can be found at: China City Statistical Yearbooks (2013–2022); China National Intellectual Property Administration (CNIPA).
